# Supplementary material for: Coronavirus risk perception and compliance with social distancing measures in a sample of young adults: Evidence from Switzerland
Source: PLoS One. 2021 Feb 19;16(2):e0247447. doi: 10.1371/journal.pone.0247447 (PMC7894933; doi:10.1371/journal.pone.0247447)
Supplement: S1 Table — (DOCX) [file pone.0247447.s001.docx]

**S1 Table. List of variables**

| Variable | Obs | Min | Max | Mean | SD | Description |
| --- | --- | --- | --- | --- | --- | --- |
| Staying at home | 493 | 1 | 5 | 4 | 0.69 | 1 = not at all *to*  5 = very strictly |
| Making exceptions | 493 | 1 | 5 | 3.54 | 0.87 | 1 = very often *to*  5 = never |
| Number of people met | 493 | 0 | 25 | 4.93 | 3.32 | In the week before the interview |
| Individual risk | 493 | 0 | 10 | 2.6 | 1.98 | 0 = not at all dangerous *to* 10 = extremely dangerous |
| Social risk | 493 | 0 | 10 | 5.52 | 1.9 | 0 = not at all dangerous *to* 10 = extremely dangerous |
| Household risk | 493 | 0 | 1 | 0.27 |  | 0 = no high-risk person in household, 1 = living with high-risk person |
| Sex | 493 | 0 | 1 | 0.65 |  | 0 = male, 1 = female |
| Age | 493 | 18 | 40 | 23.56 | 3.27 | years |
| Donation | 493 | 0 | 1 | 0.58 |  | 0 = no donation,  1 = donation |
| Social desirability | 493 | 0 | 10 | 6.16 | 1.93 | 0 = no social desirability *to* 10 = high social desirability |
| M1 Keep social distance | 493 | 1 | 5 | 4.6 | 0.64 | 1 = agree not at all *to*  5 = agree very much |
| M2 Wearing a face mask | 493 | 1 | 5 | 2.88 | 1.09 | 1 = agree not at all *to*  5 = agree very much |
| M3 Washing hands thoroughly | 493 | 2 | 5 | 4.91 | 0.34 | 1 = agree not at all *to*  5 = agree very much |
| M4 Closure of schools | 493 | 1 | 5 | 3.77 | 1.05 | 1 = agree not at all *to*  5 = agree very much |
| M5 Closure of universities | 493 | 1 | 5 | 4.15 | 0.98 | 1 = agree not at all *to*  5 = agree very much |
| M6 Closure of restaurants/ bars | 493 | 1 | 5 | 4.31 | 0.91 | 1 = agree not at all *to*  5 = agree very much |
| M7 Closure of non-food shops | 493 | 1 | 5 | 3.9 | 1.1 | 1 = agree not at all *to*  5 = agree very much |
| M8 Closure of recreational facilities | 493 | 1 | 5 | 4.18 | 0.98 | 1 = agree not at all *to*  5 = agree very much |
| M9 Closure of parks | 493 | 1 | 5 | 3.11 | 1.21 | 1 = agree not at all *to*  5 = agree very much |
| M10 Meetings of only 5 or fewer | 493 | 1 | 5 | 4.15 | 0.88 | 1 = agree not at all *to*  5 = agree very much |
| M11 Restrictions to public transport | 493 | 1 | 5 | 3.98 | 1.02 | 1 = agree not at all *to*  5 = agree very much |
| M12 Closure of boarders / travel ban | 493 | 1 | 5 | 3.82 | 1.19 | 1 = agree not at all *to*  5 = agree very much |
