# Supplementary material for: Coronavirus risk perception and compliance with social distancing measures in a sample of young adults: Evidence from Switzerland
Source: PLoS One. 2021 Feb 19;16(2):e0247447. doi: 10.1371/journal.pone.0247447 (PMC7894933; doi:10.1371/journal.pone.0247447)
Supplement: Attachment — Submitted filename: documentation_of revision.docx [file pone.0247447.s002.docx]

Dear Valerio Capraro,

First of all, we thank you and the two reviewers very much for the effort of reviewing our manuscript and for the helpful suggestions. We incorporated all of them and respond to every comment point by point as they were made.

Response to the editor

The editor recommends a list of references to be included in the paper. We thank the editor for these helpful references. We incorporated all of them into the paper, with only one exception. Particularly, the revised paper contains a new paragraph referring to this recent body of literature. This paragraph as well as the new references are marked in the marked-up copy of the manuscript.

Response to reviewer 1

1.) The reviewer suggests to include two further references: Hoh Teck Ling & Mee Chyong Ho 2020, and Moussaoui et al. 2020. Both suggestions were very helpful and we did incorporate them into the paper. The new references are marked in the marked-up copy of the manuscript. Furthermore, we point out in the summary and discussion part, that our finding replicates the results from Moussaoui et al. 2020 (see marked up copy of the manuscript). Moreover, we updated the literature review as was also suggested by the editor.
2.) We agree with the reviewer that we should be careful with causal implications. Therefore, we included a statement in the discussion part saying “Since we measured attitudes and self-reported behavior at the same time, we also cannot exclude the possibility that subjects adopted their attitudes to their self-perceived behavior instead of the other way round. Hence, the assumed causal attitude-behavior link should be investigated by non-intrusive behavioral measures in combination with a time lag of the observed behavior.”
3.) The reviewer questions the paragraph on the number of Corona deaths in the introduction. In the revised version we updated all numbers according to the official statistics in Switzerland. As it turns out, Switzerland did experience excess deaths due to the Corona Pandemic by the end of 2020. Accordingly, we wrote in the revised manuscript: “By the end of 2020, the official coronavirus death toll in Switzerland had increased to about 7400 [1], and comparing the total number of deaths for 2020 (76,231) with the number the year before (67,515) or with other years reveals that there has been a substantive excess in deaths in 2020 [2].” We hope that this takes care of the reviewer’s concerns.
4.) Concerning the statement about health problems we did provide a reference in the revised manuscript: FOPH. Weekly situation report on the epidemiological situation in Switzerland and the Principality of Liechtenstein: Week 53. Federal Office of Public Health, Switzerland 2021. The reference is also marked in the marked-up version of the manuscript.
5.) We corrected the spelling of “desirability”. Thank you very much for this comment.
6.) The uploaded version of the figures should have high resolution. This was probably not visible in the printout of the reviewers. Thanks for letting us know and we will keep an eye on it.
7.) Thank you for this comment. We harmonized the description of the sample size, which is 510.

Response to reviewer 2:

Thank you very much for your nice comments about our paper. We appreciate that you liked the manuscript. Of course, we agree with the reviewer’s comment that our study analyzes only cross-sectional data. This implies that we cannot exclude the possibility that the behavior of the participants influences their attitudes versus the other way round as we assume by our hypotheses. To make this transparent and more explicit, we included the following statement in the discussion part of revised manuscript: “Since we measured attitudes and self-reported behavior at the same time, we also cannot exclude the possibility that subjects adopted their attitudes to their self-perceived behavior instead of the other way round. Hence, the assumed causal attitude-behavior link should be investigated by non-intrusive behavioral measures in combination with a time lag of the observed behavior.” We hope that this takes care of the reviewer’s concern.
